# 

**Published:** 1903-10-24

**Authors:** 


### Publications.

JUST ISSUED. EIGHTH EDITION. Crown 8vo. 10s. 6d.

### PHARMACY, MATERIA MEDICA AND THERAPEUTICS

Is now rewritten and printed in large type, and is made a companion to the following volume. It contains an account of the action **of every drug in the B.P.**, as well as a section upon all the newest drugs, with an Introduction to the Art of Dispensing. With woodcuts, prescriptions, &c.

Also FOURTH EDITION. 1055 pages, 8vo. 16s.

### DICTIONARY OF TREATMENT.

Containing a Revised and up-to-date Article upon every Diseased Condition, whether Medical, Surgical, Gynæcological, or Ophthalmological, as well as Articles upon Poisoning, Drowning, and the various Surgical Emergencies and Accidents, arranged for Rapid Reference.

By W. WHITLA, M.A., M.D., Professor of Materia Medica, Queen's College, Belfast; Senior Physician to the Royal Victoria Hospital, &c.

The Publisher will supply both volumes, carriage paid, for **21/-**

London: HENRY RENSHAW, 356 Strand.

---

2nd Edition. Revised and Enlarged. Now Ready.
Post 8vo. pp. xv.-268, and 39 Illustrations in the text.
Cash price **4/6,** postage 4d.

### PRACTICAL TEXT-BOOK OF MIDWIFERY FOR NURSES.

By ROBERT JARDINE, M.D. Edin., M.R.C.S. Eng., F.F.P. and S. Glas.
Professor of Midwifery in St. Mungo's College, Glasgow; Physician to the Glasgow Maternity Hospital; late President of the Glasgow Obstetrical and Gynæcological Society.

"It is the best suited for its purpose of any book of the kind which we have come across. . . . . For nurses the little book is an improvement on any similar book of the kind with which we are familiar."
*The Dublin Journal of Medical Science.*

W. F. CLAY, 18 Teviot Place, Edinburgh.

---

Crown 8vo. Illustrated. Price **2s. 6d.** net.

### ANÆSTHETICS. By J. BLUMFELD,

M.D.Cantab., Senior Anæsthetist to St. George's Hospital; Anæsthetist to the Grosvenor Hospital for Women and Children, London, and to the National Dental and Metropolitan Hospitals.

*Lancet.*—"....In writing this handbook the author has been quite successful." *Australasian Medical Gazette.*—"We can strongly commend this book." *Medical Chronicle.*—"The book is one which we can heartily recommend to senior students and practitioners."

London: BAILLIÈRE, TINDALL & COX, 8 Henrietta St., Covent Garden.

---

### THE HANDBOOK OF THE OPEN-AIR TREATMENT

By Dr. CHARLES REINHARDT
(Visiting Physician to Hailey Sanatorium, Wallingford, and Stourfield Park Sanatorium, Bournemouth),
Contains Views, Descriptions, and Information respecting 30 Sanatoria in Great Britain.
Price: Cloth, 2/6; Paper, 1/- (postage 4d.)

The "HEALTH RESORT" Publishing Office, 379 Strand, London, W.C.

---

### WORKS BY DAVID WALSH, M.D.

**RONTGEN RAYS IN MEDICAL WORK.** *Third Edition.* **12s. 6d. net.** Baillière & Cox, London.

**THE HAIR AND ITS DISEASES.** 2s. 6d. net. Baillière & Cox.

**GOLDEN RULES OF SKIN PRACTICE.** 1s. net. Wright & Co., Bristol.

**AGE AND OLD AGE: a Handbook.** 2s. 6d. R. A. Everett & Co., London.

---

*NOW READY. SECOND EDITION. REVISED.*
In stiff covers, 1/- net, postage 2d. In cloth gilt, 1/6 net, postage 2d.

### THE CARE OF INFANTS.

**A Manual for Mothers and Nurses.**

By SOPHIA JEX-BLAKE, M.D.,
Member of the Irish College of Physicians, late Dean of the Edinburgh School of Medicine for Women, Lecturer on Midwifery for the University of Edinburgh, late Senior Physician to the Edinburgh Hospital for Women and Children.

Edinburgh: GEORGE A. MORTON, 42 George Street.
London; SIMPKIN, MARSHALL & Co., Ltd., and all Booksellers.

---

### WORKS BY Mr. McADAM ECCLES, M.S., F.R.C.S.,

Assistant Surgeon St. Bartholomew's Hospital, &c.

### HERNIA.

Second Edition, 1902. Price 7/6 net. Illustrated by Numerous Photographs.

### THE IMPERFECTLY DESCENDED TESTIS.

Just published. Profusely Illustrated. Price **7/6.**

London: BAILLIÈRE, TINDALL & COX.

---

Crown 8vo., price **3/6.**
With a "Plea for the State Registration of Nurses."

### MOCK NURSES OF THE LATEST FASHION.

By FREDERICK J. GANT, F.R.C.S.,
Consulting Surgeon to the Royal Free Hospital.

BAILLIÈRE, TINDALL & COX,
Henrietta Street, Covent Garden, London.

---

### MEDICAL HISTORY FROM THE EARLIEST TIMES.

By E. T. WITHINGTON, M.A., M.B. Oxon.
Demy 8vo., over 400 pp., with two Plates, cloth gilt, **12/6 net.**

"One of the best attempts that has yet been made in the English language to present the reader with a concise epitome of the history of medicine, and as such we very cordially commend it."
*Glasgow Medical Journal.*

THE SCIENTIFIC PRESS, Ltd.,
28 and 29 Southampton Street, Strand, London, W.C.

### NOTICE TO CORRESPONDENTS.

All MSS., Letters, Books for Review, and other matters intended for the Editor should be addressed The Editor, "The Hospital," The Hospital Buildings, 28 & 29 Southampton Street, Strand, W.C.

The Editor cannot undertake to return rejected MS., even when accompanied by stamped directed envelope.

**Notice to Advertisers and Subscribers.**

All Advertisements, Orders for Copies of The Hospital, and Business Communications should be addressed to **THE MANAGER (Not to the Editor)**, The Hospital, 28 & 29 Southampton Street, Strand, London, W.C.

The Cost of Subscription, post free, is as follows:—Per week, 3½d.; per quarter, 3s. 9d.; per half-year, 7s. 6d.; per annum, 15s. Foreign and Colonial:—Per annum, 19s. 6d., payable, in all cases, in advance.

**NOTICE.—Vol. XXXIV. of "The Hospital" (April 1903 to September 1903), in handsome cloth binding, will shortly be on sale at the office of this Journal, price 7s. 6d. Cases for binding the Numbers contained in this Volume 1s. 6d. Index to Vol. XXXIV., 2½d., post free.**

**The Monthly part for October is now ready. Price 1s., by post, 1s. 3d.**

### SCRAPS AND GLEANINGS.

At an inquest held recently at Knutsford Gaol on a woman aged 62, it transpired that since she was 12 years of age she had only been a free woman for eight years.

The organisers of the Blackpool Hospital Saturday this year have every reason to be satisfied with the result of their efforts, no less a sum than £300 having been paid over to the Victoria Hospital, being the amount collected on that day, after deduction of expenses.

The Ladies' Hospital Collection Committee have also had a gratifying success in Bury St. Edmund's this year, a total sum of £104 odd having been raised, as against £80 odd last year. Of the former amount £75 resulted from the fête in Culford Park, kindly lent by the Earl and Countess Cadogan.

The area of the site of the new workhouse and infirmary at Rothwell Haigh is 18 to 19 acres; the present accommodation is for 450, so that there is ample room for future extensions. The infirmary is a complete building in itself, and is connected with the main building, which is the workhouse proper, by a covered corridor. The total cost of all the buildings, including purchase of land, is £85,850.

Despite the decision of the governors of the East Suffolk and Ipswich Hospital, at their meeting held on June 24th last, when Dr. Bartlet's offer to build, in a central part of the town, an out-patients' department attached to the hospital was accepted, the special committee which was then appointed to consider Dr. Bartlet's offer have decided that the new out-patients' department should be built within the hospital grounds, and Dr. Bartlet consenting to this modification of his original scheme, it has been determined that the new out-patients' department, which he has so generously offered to provide, shall be built in the south-east corner of the hospital grounds, and not in the town as originally intended.

### THE STUDENT OF MEDICINE.

### APPOINTMENTS.

**Anthony A. Bowlby**, F.R.C.S., Surgeon to St. Bartholomew's Hospital. **R. H. G. Bruce**, M.B., M.S.Aberd., Certifying Factory Surgeon for the Friockheim District, county Forfar. **John Costelloe**, M.D., B.S., Medical Officer to the Balranald Hospital, New South Wales. **Robert T. Edwards**, M.R.C.S.Eng., L.R.C.P.Lond., Resident Medical Officer to the Aberystwyth Infirmary and Cardiganshire General Hospital. **H. A. Ellis**, M.B., Ch.B.Dub., Health Officer at Coolgardie, West Australia. **A. Courtenay Evered**, L.R.C.P.&S.Edin., Health Officer at Albany, West Australia. **Robert E. Kane**, L.R.C.P.&S.Edin., Medical Officer at Taroom, Queensland. **A. D. Kennedy**, M.B. Ch.B.Glasg., Certifying Factory Surgeon for the Ballachulish District, County Argyll. **Wilfred Knight**, M.B., L.R.C.P.E., Resident Medical Officer to the Durban Hospital, Natal. **Thomas Hillhouse Livingstone**, M.D., F.R.C.S.Edin., Medical Officer to the Newcastle-on-Tyne Branch of the National Society for Prevention of Cruelty to Children. **G. W. Mould**, M.R.C.S.Eng., Consulting Medical Officer to the Royal Asylum, Cheadle, and Superintendent of the Welsh houses connected therewith. **P. G. Mould**, M.R.C.S., L.R.C.P., Second Assistant Medical Officer to the Royal Asylum, Cheadle. **William L. Mullen**, M.B., Ch.B.Melb., Deputy Medical Superintendent of the Kew Lunatic Asylum, Victoria. **Richard A. O'Brien**, M.B., B.S.Melb., Health Officer for the Port of Brisbane. **J. S. Poole**, M.D.Canada, Clinical Assistant to the Chelsea Hospital for Women. **R. H. Sankey**, M.A.Oxon., M.R.C.S., L.R.C.P.Lond., House Physician to the Radcliffe Infirmary, Oxford. **Walter Scowcroft**, M.R.C.S., L.R.C.P., Resident Superintendent to the Royal Asylum, Cheadle. **Patrick F. Shanahan**, M.B., B.S.Adel., Health Officer for Arltunga, South Australia. **H. H. Stiff**, M.B., B.C.Cantab., M.R.C.S., L.R.C.P.Lond., Certifying Surgeon under the Factory and Workshops Act for the Bury St. Edmunds District. **John Sutcliffe**, M.R.C.S., L.R.C.P., Senior Assistant Medical Officer to the Royal Asylum, Cheadle. **Edwin R. Thomas**, L.R.C.P.&S.E., L.F.P.&S.G., Medical Officer of Health for the Borough of Beaumaris. **Eric M. Thomson**, M.A., M.B., Ch.B.Aberd., Assistant Medical Officer, Government Lunatic Asylum, Kingston, Jamaica. **W. H. Unwin**, M.B., F.R.C.S., Clinical Assistant to the Chelsea Hospital for Women. **A. T. Waterhouse**, M.B., B.Ch.Oxon., L.R.C.P., M.R.C.S., House Surgeon to the Radcliffe Infirmary, Oxford. **Samuel West**, M.D.Oxon., F.R.C.P., Physician to St. Bartholomew's Hospital. **James Wood**, M.R.C.S.Eng., L.R.C.P.Lond., L.D.S., Honorary Dental Assistant Surgeon to the Bolton Infirmary and Dispensary.

### "The Hospital" Institutional Library.

"Hospitals and Asylums of the World." 4 Vols., with a Portfolio of Plans, complete £12 12s.

"Burdett's Hospitals and Charities, 1903." 5s. net.

"Burdett's Official Nursing Directory, 1903." 3s. net.

"Cottage Hospitals, General, Fever, and Convalescent." 10s. 6d.

"Uniform System of Accounts for Hospitals and Public Institutions." New Edition. 4s. net.

"Hospital Expenditure: The Commissariat." 2s. 6d.

"A Legal Handbook for the Use of Hospital Authorities." 2s. 6d.

"The Cottage Hospital Case Book or Register of Patients." 10s. and 12s. 6d.

All these are published by the Scientific Press, Ltd., and may be obtained through any bookseller, or direct from the publishers, 28 and 29 Southampton Street, Strand, London, W.C.

**PRACTICAL HINTS ON DISTRICT NURSING.**
By AMY HUGHES.
Crown 8vo. cloth, 1/- post free.

**FEVERS AND INFECTIOUS DISEASES:**
Their Nursing and Practical Management.
By WILLIAM HARDING, M.D.Edin., M.R.C.P.Lond.
Crown 8vo. cloth, 1/- post free.

**DISTRICT NURSING ON A PROVIDENT BASIS.**
By JAMIESON B. HURRY, M.D.
Crown 8vo., cloth gilt, 2/- post free.

**THE POCKET CASE BOOK FOR DISTRICT AND PRIVATE NURSES.**
By A PHYSICIAN.
Demy 16mo. cloth, 1/- post free.

*Of all Booksellers, or of*
THE SCIENTIFIC PRESS, LTD., 28 & 29 Southampton Street, Strand, London, W.C.

### Useful Handbooks for Mothers and Nurses.

**THE MOTHER'S HELP AND GUIDE TO THE DOMESTIC MANAGEMENT OF HER CHILDREN.** By P. MURRAY BRAIDWOOD, M.D.
Second Edition, with Addenda. Crown 8vo., cloth, **2/-** post free.
"Mothers and nurses will find in Dr. Braidwood's pages a very large amount of well-selected information on the management of children both in health and sickness."—*Lancet.*

**DIET IN SICKNESS AND IN HEALTH.** By Mrs. ERNEST HART. With an Introduction by Sir HENRY THOMPSON, F.R.C.S., M.B.Lond.
Fourth Thousand. Demy 8vo., Illustrated, cloth gilt, **3/6** post free.
"We have perused this book with great pleasure, and feel sure that many will find in it much to lighten the days of those whose digestion is enfeebled."—*The Hospital.*

**ART OF FEEDING THE INVALID.** By a MEDICAL PRACTITIONER and a LADY PROFESSOR OF COOKERY.
New and Popular Edition. Demy 8vo., cloth, **1/6** post free.
"A new edition of this valuable work is very welcome, and we heartily commend it to all who desire reliable and practical instruction in sick cookery."—*Nursing Notes.*

**SPINAL CURVATURE AND AWKWARD DEPORTMENT: THEIR CAUSES AND PREVENTION IN CHILDREN.** By Dr. GEORGE MÜLLER, Professor of Medicine and Orthopædics, Berlin. English Edition, edited and adapted by RICHARD GREENE, F.R.C.P.Edin.
Crown 8vo., cloth gilt, with Plates and Illustrations. **2/6** post free.
"It might be read by most parents and schoolmasters with advantage, for it sets out clearly the causes of common deformities and how to avoid them in plain, simple language."
*British Medical Journal.*

*Of all Booksellers, or of*
THE SCIENTIFIC PRESS, LIMITED, 28 & 29 Southampton Street, Strand, London, W.C.

### THE PRIVATE NURSE'S OWN NOTE-BOOK.

*Just Published.* By SISTER EVA (with photo). Author of "Scenes in the Life of a Nurse," &c. Price 6d. post free from BEMROSE & SONS, Derby.

A most useful little work for the help of Private Nurses, in which all particulars of cases can be easily recorded. The volume also contains tear-off pages for expenses incurred at cases, "Dainty Dishes for Invalids," &c. Designed to last for at least a year. An invaluable reference book.

### The Probationer's Library.

### THE NURSING PROFESSION:

### HOW AND WHERE TO TRAIN.

**Edited by SIR HENRY BURDETT, K.C.B.**

An Indispensable Guide to the would-be Nurse. Price **2/-** net; **2/4** post free.

---

### Three Admirable Nursing Handbooks.

### A HANDBOOK FOR NURSES.

By Dr. J. K. Watson. Price **5/-** net; **5/4** post free.

Very full and up to date.

### NURSING: ITS THEORY AND PRACTICE.

By Dr. Percy G. Lewis. Price **3/6** post free.

A complete Guide for the Medical, Surgical and Monthly Nurse.

### NURSING: ITS PRINCIPLES AND PRACTICE.

By Isabel Hampton. Price **7/6** net; **7/10** post free.

A valuable Guide to Nursing by a well-known American Matron.

### ELEMENTARY ANATOMY AND SURGERY FOR NURSES.

By W. McAdam Eccles, F.R.C.S. Price **2/6** post free.

A clear and concise instructor

### ELEMENTARY PHYSIOLOGY FOR NURSES.

By Dr. C. F. Marshall. Price **2/-** post free.

The subject is completely and clearly explained.

### PRACTICAL GUIDE TO SURGICAL BANDAGING AND DRESSINGS.

By Wm. Johnson Smith, F.R.C.S. Price **2/-** post free.

### THE NURSE'S DICTIONARY OF MEDICAL TERMS AND NURSING TREATMENT.

Compiled by Honnor Morten. Price **2/-** post free.

An invaluable pocket-book for Nurses.

*These books are obtainable of all Booksellers, and are published by*
THE SCIENTIFIC PRESS, LIMITED, 28 & 29 Southampton Street, Strand, London,
*who will send their latest Catalogue of Nursing Text-books post free to any Nurse on application.*

Standard Works | **J. & A. CHURCHILL'S** | Latest Editions

**Books for Nurses.**

**NURSING.**

**Nursing, General, Medical and Surgical, with an Appendix on Sick Room Cookery.** By Wilfred J. Hadley, M.D., F.R.C.P., F.R.C.S., Physician and Pathologist to the London Hospital, Lecturer to Nurses at the London Hospital Nursing School . . . . 3s. 6d.

**MEDICINE.**

**Lectures on Medicine to Nurses.** By Herbert E. Cuff, M.D., F.R.C.S., Medical Superintendent, North Eastern Fever Hospital, London. Fourth Edition. With 29 Illustrations . . . . . . . 3s. 6d.

**INFANT FEEDING.**

**The Natural and Artificial Methods of Feeding Infants and Young Children.** By Edmund Cautley, M.D., F.R.C.P., Physician to the Belgrave Hospital for Children; late Casualty Physician and Assistant Demonstrator of Physiology, St. Bartholomew's Hospital. Second Edition. 7s. 6d.

**DIET.**

**Diet and Food considered in relation to Strength and Power of Endurance, Training and Athletics.** By A. Haig, M.D., F.R.C.P. Fourth Edition. 2s.

**MIDWIFERY.**

**A Short Practice of Midwifery for Nurses.** By Henry Jellett, M.D. (Dub Univ.), F.R.C.P.I., Ex-Assistant Master, Rotunda Hospital, Dublin, Extra Examiner in Midwifery and Gynæcology, Royal College of Physicians of Ireland, Extern Examiner in Midwifery, Royal University of Ireland. With 67 Illustrations . . . . . 6s.

**MONTHLY NURSING.**

**A Short Manual for Monthly Nurses.** By C. J. Cullingworth, M.D., F.R.C.P., Obstetric Physician to St. Thomas's Hospital. Revised with the assistance of M. A. Atkinson, Matron of the General Lying-in Hospital, London. Fifth Edition. 1s. 6d.

**HEALTH OF A WIFE.**

**Chavasse's Advice to a Wife on the Management of her Own Health.** Fourteenth Edition. By Fancourt Barnes, M.D., Consulting Physician to the British Lying-in Hospital . . . . 2s. 6d.

**HEALTH OF CHILDREN.**

**Chavasse's Advice to a Mother on the Management of her Children.** Fifteenth Edition. By George Carpenter, M.D., Physician to the Evelina Hospital for Children . . . . . . 2s. 6d.

**ANTISEPTICS.**

**Lessons in Disinfection and Sterilisation;** an Elementary Course of Bacteriology, with a Scheme of Practical Experiments. By F. W. Andrewes, M.D., F.R.C.P., Lecturer on Pathology at St. Bartholomew's Hospital. With Illustrations . 3s. net. [*Just Published.*

**SURGERY.**

**A Manual of Minor Surgery and Bandaging.** By Christopher Heath, F.R.C.S., and Bilton Pollard, F.R.C.S., Surgeon to University College Hospital. Twelfth Edition. With 195 Illustrations. 6s. 6d.

**HYGIENE.**

**The Elements of Health:** An Introduction to the Study of Hygiene. By Louis C. Parkes, M.D., D.P.H.Lond., Lecturer on Public Health at St. George's Hospital. 3s. 6d.

**DICTIONARY.**

**A Short Dictionary of Medical Terms.** By R. G. Mayne, M.D., LL.D. . 2s. 6d.

7 GREAT MARLBOROUGH STREET, LONDON.

